# Current and future advances in practice: practical management of hand osteoarthritis

**DOI:** 10.1093/rap/rkaf093

**Published:** 2025-09-19

**Authors:** Fiona E Watt, Donna L Kennedy, Matthew D Gardiner, Tonia L Vincent

**Affiliations:** Department of Immunology and Inflammation, Imperial College London, London, UK; Centre for Osteoarthritis Pathogenesis, Kennedy Institute of Rheumatology, NDORMS, University of Oxford, Oxford, UK; Rheumatology Department, Imperial College Healthcare NHS Trust, London, UK; Therapies Department, Imperial College Healthcare NHS Trust, London, UK; Centre for Osteoarthritis Pathogenesis, Kennedy Institute of Rheumatology, NDORMS, University of Oxford, Oxford, UK; Department of Plastic Surgery, Wexham Park Hospital, Frimley Health NHS Foundation Trust, Slough, UK; Centre for Osteoarthritis Pathogenesis, Kennedy Institute of Rheumatology, NDORMS, University of Oxford, Oxford, UK; Rheumatology Department, Imperial College Healthcare NHS Trust, London, UK

**Keywords:** hand, osteoarthritis, treatment, drug, therapy, exercise, splint, surgery, flare, pain

## Abstract

The high-quality management of hand osteoarthritis (OA) is crucial for improving the daily lives of people with the condition. The 2022 National Institute for Health and Care Excellence (NICE) guidelines for OA management emphasize education, weight advice and physical activity as foundational management strategies, although hand OA requires specific, tailored approaches. While there can be a focus on pharmacological treatments, non-drug interventions such as education, hand exercises, splinting, joint protection and ‘offloading’ should be prioritized. Pharmacological options including topical NSAIDs are recommended before considering oral medications or intra-articular steroid injections. Although no disease-modifying therapies exist, many symptom management strategies are available. In cases where conservative treatments fail, surgical interventions such as joint fusion, trapeziectomy or arthroplasty are options. The latest insights into OA management based on collective clinical expertise as well as research are outlined. This is underpinned by a current view of pathogenesis, with the hope of enabling a positive consultation incorporating current, accurate information about hand OA. Addressing concerns, myth-busting and using non-negative language are important for activation and empowerment.

Key messages

- Just because it is common, this does not mean hand osteoarthritis is inevitable or untreatable, or not important to the individual or the healthcare provider.
- A multimodal approach is important: communication and good quality person-centred advice is key, supporting positive, evidence-based care; it is osteoarthritis, not just ‘it is not rheumatoid arthritis (RA)’.
- Advice (including accurate information) from a clinician can be a transformative therapy: do not assume anyone other than you will do this.
- Mechanical load (and offload) is one of the most important factors in the evolution, flare and progression of hand osteoarthritis and should be considered therapeutically.
- There is always something we can do, including (but not starting with) drugs or surgery. It is important to implement the treatments we have.

## Clinical importance of hand OA: impact and current state

Symptomatic hand osteoarthritis (OA) is the most common form of OA, affecting 22% of adults >50 years of age (16% males, 28% females). It causes joint pain, stiffness and reduced hand function, impacting independence, confidence and work [1–3]. Its effects on physical function can exceed those of rheumatoid arthritis (RA), which has far more treatment options [4]. In its early, inflammatory phases, hand OA may sometimes be hard to differentiate from inflammatory arthritis, or in later stages, due to pain and functional challenges. At any point, it may pose challenges in management.

Hand OA is more common in women, particularly after menopause. In secondary care, the female:male ratio may be at much as 9:1, possibly due in part to sex-specific differences in severe symptoms or multiple joint involvement [5, 6]. However, many with hand OA experience mild symptoms that respond to self-management, often managed in primary care or never seeking healthcare. Surgical pathways may see more patients with persistent pain at the base of the thumb, whereas non-surgical pathways are more likely to see those with early swelling of finger joints or multiple finger joint involvement. To provide a comprehensive view, we include insights from a multidisciplinary author team.

There is an unmet need for effective treatments for hand OA, both to slow progression and target pain. Hand OA has historically received less attention than hip and knee OA, perhaps relating to a less well-articulated economic case, leading to fewer clinical trials and limited progress in pharmacological options. While clinical guidelines (summarized in Table 1) have mainly focused on large joints or are joint agnostic. Adapting general OA guidance to hand OA remains a management challenge.

**Table 1.** Summary of current international guidance relevant to the management of hand OA.

Body/institution	Title of guidance	Link to guidance	Year published [reference]
National Institute for Health and Care Excellence (NICE)	Osteoarthritis in over 16s: diagnosis and management [NG226]	https://www.nice.org.uk/guidance/ng226	2022 (update) [7]
European Alliance of Associations for Rheumatology (EULAR)	2018 update of the EULAR recommendations for the management of hand osteoarthritis	https://ard.bmj.com/content/78/1/16	2018 [8]
Osteoarthritis Research Society International (OARSI)	OARSI guidelines for the non-surgical management of knee, hip and polyarticular osteoarthritis 2019	https://www.oarsijournal.com/article/S1063-4584(19)31116-1/fulltext	2019 [9]
American College of Rheumatology (ACR)	2019 American College of Rheumatology/Arthritis Foundation guideline for the management of osteoarthritis of the hand, hip, and knee	https://acrjournals.onlinelibrary.wiley.com/doi/10.1002/acr.24131	2019 [10]
BSSH (NICE accredited) Evidence for Surgical Treatment (BEST)	Evidence-based management of thumb base osteoarthritis	https://www.bssh.ac.uk/professionals/best_guidelines.aspx	2023 [11]
Results from a European consensus study	Quality indicators for hand osteoarthritis care	https://www.oarsiopenjournal.com/article/S2665-9131(25)00014-7/fulltext	2025 [12]

## Scope and remit of this review

This review shares the clinical expertise of those with extensive experience in managing hand OA. It provides insights and ‘clinical pearls’ based on practical experience of what works (and what doesn’t). Research evidence is also provided, but this is not a systematic literature review. Where views are based on anecdotal experience, this is noted. Its scope is summarized in Table 2. The number and location of affected joints are key to management, considered throughout. While focused on idiopathic hand OA, most of the content also applies to secondary OA, such as post-traumatic OA. In our clinical experience, these are a small minority of cases: the disease appears clinically indistinguishable (though often focused on one joint) and current treatment options are no different.

**Table 2.** Scope of this review.

Area	What is included?	What is not included?
Affected joint sites	Hand osteoarthritis (OA)—one to many affected joints in the hand These include all commonly affected sites in the hand—interphalangeal (IP) joint (both proximal and distal), base of thumb, including carpometacarpal (CMC) and scaphotrapeziotrapezoidal (STT) and any combination of these	Wrist OA Other non-hand OA
Focus of review	Clinical management of hand OA The importance of communicating the diagnosis of OA and how to do this well The importance of detection of relevant clinical aspects and concurrent conditions relevant to management Key aspects of current understanding of pathogenesis, aetiology and natural history with a view to management	Making a diagnosis of hand OA Disease features or routine clinical assessment Detailed aspects of aetiopathogenesis, including molecular pathogenesis (for all, see Marshall *et al.* [2])
Types and routes of clinical presentation	All hand OA presentations to a clinician (one joint to many) Particular focus on those with moderate to severe symptoms not responding to first-line management seen in secondary care, i.e. ‘difficult-to-treat’ hand OA	Management considerations specific to secondary forms of OA

We hope this article will be of interest to a wide range of practitioners including occupational therapists and physiotherapists, specialist nurses, general practitioners, rheumatologists and orthopaedic surgeons, including doctors in training.

## Natural history and aetiology of hand OA with a view to its management

Understanding the natural history of hand OA is crucial for discussions on ‘what to expect’ as well as management. The disease typically progresses through recognizable phases [13]. In our experience, ‘inflammatory’ with synovitis and erosion, ‘repair and remodelling’ with joint shape changes and ‘resolving/quiescent’ with bony deformity, stiffness and less pain are all frequently discernible. Different joints may be in different phases. The term ‘inflammatory, erosive osteoarthritis’ suggesting a discrete clinical entity is misleading, as inflammation and erosion are present in many patients [14–16]. However, whether erosions are universally or frequently present remains controversial [2, 17]. Severe multijoint erosive joint disease is difficult to treat, so identifying this phenotype may help focus on a group with particularly poor outcomes. The time to reach the ‘quiescent’ phase varies, but many patients improve in 2–5 years, especially females. This can vary, with some people having active disease for more than a decade.

Hand OA can affect one or more ‘rows’ out of the distal interphalangeal (DIP) joint, proximal interphalangeal (PIP) joint or base of the thumb [2]. Other hand joints and the knee may be affected (due in part to shared genetic risk), although more generalised OA is clinically far more unusual. Isolated DIP joint OA tends to have a better prognosis. It is common for patients to worry about OA spreading, but reassurance and information on natural history can help reduce anxiety.

Flares, well recognized in inflammatory arthritis, also occur in many patients with hand OA and last a few days, although evidence quantifying this is lacking. In our experience, mechanical triggers are common. Helping to recognize these (and any related triggers) and developing strategies for their self-management and prevention is useful, although the impact of this on disease progression is unclear.

During consultations, we recommend identifying modifiable and non-modifiable risk factors for progression and flare. Prior hand trauma, mechanical overload and hypermobility at the base of the thumb increase risk. Mechanical overload through occupation (cleaner, caregiver, artist) or recreational activities (do-it-yourself, gardening) is not unusual. Menopausal timing and changes in hormonal treatments should be documented [e.g. hormone replacement therapy (HRT), oral contraceptive pill, gonadotropin-releasing hormone analogues, aromatase inhibitors, ovulation therapies, anti-androgens etc]. Although not immediately intuitive, the role of weight management in modulating systemic and joint-based inflammation, including in the hand, should not be underestimated. Loss of weight is associated with a reduction in low-grade systemic inflammation [18, 19]. Psychological factors like anxiety, depression and low self-efficacy also influence pain levels in hand OA [20].

## Pathogenesis of hand OA with a view to its management

OA at all joints is driven by an active cellular response to mechanical stress (paralyzed joints, including hand joints, do not get OA) [21]. Mechanically induced signalling drives inflammatory pathways that contribute to pain and tissue damage (so-called mechanoflammation) [22]. Mechanical stress also drives pro-regenerative pathways that may contribute to remodelling of bone, cartilage and other soft tissues. There is a fundamental difference between this type of inflammatory response and that seen in inflammatory arthritis, likely explaining why trials of established RA treatments have mostly failed in OA (reviewed in [23]). On the other hand, mechanically ‘offloading’ OA joints experimentally (mainly large joints) ameliorates disease in animals and in humans [24, 25].

OA affects all the tissues of the joint and several of these may contribute to pain, stiffness and deformity. Although articular cartilage is aneural, it is a recognized source of pro-nociceptive and inflammatory molecules that can drive pain sensitization and cartilage degradation [26]. Bony and ligamentous remodelling are also evident on examination and may be sources of pain, including at the enthesis [27]. Heberden’s nodes at the DIP joints are not infrequently preceded by synovial cysts that can occasionally rupture to the exterior. Drainage is generally avoided so as to reduce the chance of introducing infection into the joint cavity. The role of the synovium in the pathogenesis of OA remains controversial. Synovial inflammation is common and associated with joint pain in most individuals [14]. Treatment of synovitis when active, e.g. by non-steroidal anti-inflammatory drug (NSAID) treatment, steroid injection or offloading, may lead to symptom improvement and a reduction in swelling that allows exercises to maintain and improve joint mobility (see below).

Our knowledge of molecular pathogenesis in hand OA has been underpinned by recent genome-wide association studies (GWASs) that have identified a number of putative genes with a causal role in disease [28, 29]. One such gene is retinaldehyde dehydrogenase 2 (*ALDH1A2*), encoding an enzyme that controls retinoic acid levels. Polymorphic variants in this gene are common in the population and lead to reduced levels of retinoic acid, which is associated with higher levels of mechanoflammation in hand OA cartilage and worse hand disease [29, 30].

## Evidence-based management approaches

Table 1 provides information and links to various international guidance. The evidence-based, independently collated general advice from the National Institute for Health and Care Excellence (NICE) on management of OA was updated most recently in October 2022 [7] and remains a cornerstone of management for OA at any site for many. The current iteration removed some management options and provoked controversy in doing so. NICE guidelines are not joint specific, so advice needs to be tailored to management of hand OA specifically. In our experience, many physicians are not familiar with NICE guidance on OA and seldom follow its algorithms; for instance, by jumping to drug-based options before advocating for non-drug options or prescribing first-line oral rather than topical options.

In 2018, EULAR published hand OA management guidance [8]. Aspects of note were caution around evidence for hand intra-articular injections and splinting (though less could be said about these interventions at IP joints). The British Society for Surgery of the Hand (BSSH) has also published guidance specifically relating to the base of the thumb [11] (Table 1), but most other current guidance does not relate specifically to the hand. Hand OA is generally underrepresented in clinical trials and data are lacking that are generalizable across joint sites (e.g. nearly all injection and splinting trials are of the base of the thumb).

Here we discuss our current approaches in the light of current evidence (or lack thereof) and other refinements based on collective experience.

## ‘What works?’: a clinical perspective

We have compiled a ‘top 10’ key tips for what works in practice in our clinical experience (Table 3). We expand on some of these areas further in the sections below, focusing on certain modalities in management. Table 4 summarizes publicly accessible resources to support the management of hand OA.

**Table 3.** Top 10 practical tips for management of hand OA.

Top 10 tips	Comments	Tips to try	Tips to avoid
1. Make a clear diagnosis of hand OA	Self-management is a cornerstone of OA treatment. People cannot manage their condition if they do not know what they have. If there is uncertainty about the diagnosis leading to investigations, make sure to have a follow-up conversation (by phone or in person) to provide the diagnosis and information.	Use the term ‘osteoarthritis’. Make a diagnosis, even if the person is young or it is early stage. Focus on what it is rather than what it is not (e.g. inflammatory arthritis).	Try to avoid synonyms (e.g. OA, arthritis, wear and tear), as this can be confusing. Do not request imaging to diagnose hand OA with appropriate history and clinical features. If imaging has been done, talk about the features using non-negative terms (avoid ‘bone on bone’ or ‘degeneration’).
2. Give positive, accurate messages about what to expect from OA	Having a good-quality conversation and answering questions has been shown to help self-management. Hope, understanding and interest are good therapeutics.	Educate that not all OA gets worse (and quite a lot gets better). Support consultations with written information (e.g. Versus Arthritis leaflet^a^) but not at the expense of the conversation.	Avoid rushing in talking to people about their diagnosis and explaining the natural history—what to expect is an important part of management.
3. Explain that OA is mechanically driven by an active cellular process	People understanding that the science behind OA suggests their joint is not just an inert surface wearing out helps them to understand there are things they can actively do to help manage their condition. Knowing that there is the possibility of modification of the disease course and symptoms through self-management is motivating and evidence-based.	Do say that mechanical factors are important in their joint condition, both in its development and in their symptoms getting worse (or just as importantly, better).	Do not use the term ‘wear and tear’.
4. Consider if there is another concurrent condition (and treat it)	Vitamin D insufficiency affects muscle and so can impact pain indirectly. It is common in the groups we see. It is not an evidence-based treatment for OA symptoms or structure, but insufficiency should be addressed for general musculoskeletal heath.	Remain vigilant and treat the following conditions that can coexist and may be more common in this group: Flexor tendinopathy Carpal tunnel syndrome De Quervain’s tenosynovitis Calcium pyrophosphate deposition disease (CPPD) Gout/hyperuricaemia Type II diabetes mellitus (DM)/metabolic syndrome Fibromyalgia Polymyalgia rheumatica Inflammatory arthritis	Avoid missing symptoms or signs not in keeping with joint-based pathology: Restriction of range or digital pain *vs* only joint involvement Disproportionate issues with fine motor tasks; sensory symptoms; whole hand pain and/or dropping things suggesting neural issues Excessive inflammatory flares or atypical joint distribution (wrist or MCP joint predominance suggesting inflammatory arthritis) Myalgia
5. Advise hand exercises to all people with hand OA	There is an evidence base for exercise modifying symptoms and functional progression and their use at all stages of disease.	Routinely advocate and show patients hand exercises yourself, including range-of-motion exercises (you do not need to be a hand therapist to do this). Signpost resources and links (Table 4).	Do not exclude a short conversation on exercise because you are referring to a hand therapist. Do not forget to advocate for general exercise as well as joint-based exercises.
6. Identify ways to mechanically offload the hand joints	There is evidence that addressing excessive joint loading helps pain and might modify disease. What this means may be quite individualized. Some people will be ‘working through the pain’ or putting inadvisably high loads on the hands (osteoarthritic joints are less resilient)—remember to ask about this. Identifying personal mechanical triggers (even occasional ones) is useful.	Take a history of relevant occupational and leisure or homeworking activities that might be culprits. Help the person to think through how personal triggers/drivers can be modified: whether they can be stopped, reduced or done differently. Splinting of various kinds can sometimes provide useful ‘offload’, as can pacing and joint care advice.	Make clear this is not advice to avoid use of the hands: ongoing use is essential and underuse can lead to stiffening of joints or deconditioning of hand muscles. Do not forget to advise on weight loss and general exercise (likely beneficial, even in the hand due to systemic effects).
7. Consider analgesics when there is pain uncontrolled by other means	Do not use analgesics as a first-line treatment, but also emphasize that if they have moderate, severe or persistent pain despite other strategies, regular analgesia is justified and evidence-based.	Remember to advocate for topical NSAIDs first (awkward to use sometimes, but has a strong evidence-base that patients often are not aware of). Suggest blood pressure and renal function surveillance in the minority on regular coxibs or NSAIDs.	Do not rule out acetaminophen for multiple joint disease where there appears to be a clinical response as part of an analgesic ladder (we lack data for hand OA). Do not forget to recommend proton pump inhibitors for those using over-the-counter NSAIDs.
8. Think about the life course and sex-specific factors	There is increasing evidence to support a role for menopause and sex hormones in symptomatic hand OA. A reproductive history including a history of menopause symptoms can set any joint-based symptoms in context: menopause is associated with increased likelihood of musculoskeletal pain.	Remember to take a reproductive history: many patients are peri- or postmenopausal women. Ask about last/final menstrual period and use of HRT. Was it stopped abruptly? (this should be avoided). Was there a history of issues with menstrual periods, endometriosis etc? Encouraging a discussion with a prescriber such as a general practitioner if HRT is a consideration may be helpful. If HRT is ever stopped, advise to taper this over as long a period as possible before stopping (proportionate to how long they have taken it for and considering if there are tolerability or safety concerns).	In women, do not forget to ask about symptoms of menopause (or for men, andropause), as well as hot flashes, myalgia, disturbed sleep, fatigue, anxiety, anger or mood swings, poor concentration and urogynaecological symptoms such as vaginal discomfort and loss of libido, are all common.
9. Treat a flare of OA like a soft tissue injury	In a flare, tissues are generally responding to aberrant mechanical loading with inflammation, symptoms and signs of which are usually apparent. Just like an injury, most people will identify a likely precipitant.	Adapt management from the well-known ‘RICE’ acronym: rest (offload), ice (or warmth, whatever is most helpful), compression (hand compression garments, splints), elevation (ease inflammation with use of topical or oral NSAIDs).	Do not forget to try to identify the precipitant with the person, with the aim of reducing further flare severity/frequency.
10. Avoid suggesting a ‘single solution’	Controlling symptoms is often multifaceted and management should be layered on. There is always something to try, although what is effective for the person may change over time. Success is often a multimodal approach that is personalized and led by the person.	Involve others in the multidisciplinary team as necessary and empower the person and their general practitioner to try different approaches. Encourage good sleep hygiene, general exercise and managing stress and anxiety, they will all help pain and well-being. Remind/remember things that worked previously.	Do not use steroid injections in place of all other care. Do not treat severe OA as inflammatory arthritis, i.e. do not use hydroxychloroquine or anti-TNF therapy.

a See Table 4 for details.

**Table 4.** Summary of publicly available management resources for hand OA, including information and hand exercises.

Body/institution/group	Title of resource	Link to resource	Year published
Versus Arthritis	Osteoarthritis of the hand and wrist—leaflet, video of exercises	https://versusarthritis.org/about-arthritis/conditions/osteoarthritis-of-the-hand-and-wrist/ General hand exercises including video: https://versusarthritis.org/about-arthritis/exercising-with-arthritis/exercises-for-healthy-joints/exercises-for-the-fingers-hands-and-wrists/	2021
Jigsaw-E (Keele University)	Osteoarthritis of the hand—educational videos	https://jigsaw-e.com/delivery-toolkit/hand-osteoarthritis-education/	2019
Keele University	Osteoarthritis of the hand—leaflet	https://www.jigsaw-e.com/wp-content/uploads/2019/10/OA-Hand-Leaflet-v.0.10-02.02.18-LC-FINAL.pdf	2019
OTTER-II (University of Southampton)	Exercises and pacing	https://bmjopen.bmj.com/content/9/10/e028342.long#DC1 Supplementary appendix with OTTER-II exercises to download: bmjopen-2018-028342supp003.pdf	2019
TOPS study (University Hospitals of Derby and Burton NHS Foundation Trust)	Downloadable patient exercise and education sheets in multiple languages	https://www.thetops.study/tops-info-sheets	2023
British Society for Surgery of the Hand (BSSH)	Basal thumb OA	https://www.bssh.ac.uk/patients/conditions/24/basal_thumb_arthritis [11]	
Pulvertaft Hand Centre (Derby)	Osteoarthritis at the base of thumb (surgical options)	https://www.uhdb.nhs.uk/download/osteoarthritis-base-of-thumb-1163v4pdf.pdf? ver=84304&doc=docm93jijm4n3214	

## Provision of advice and information

A conversation providing high-quality information is essential to support effective management. Language should be carefully considered to be accurate and positive in encouraging self-management (Table 3). Myth-busting and discussing the future (the disease’s natural history) are productive and usually reassuring.

Epidemiological studies suggest that ≈45% of people with knee OA have resolving (non-progressive) pain [31]. Research suggests that the same may be true for the hand [32]. Like RA, earlier-stage disease could potentially be more modifiable, so it is useful to have these conversations early on. It is important for people to understand that progression of the disease is not inevitable—to a surgeon or an unusable joint—and that their condition is potentially modifiable (although we lack drugs that do this). Using terms like ‘wear and tear’ may minimize the condition, make people passive bystanders or infer this is entirely ageing-related or inevitable (neither true nor helpful). Even in the hand, the opportunity to talk about weight control and loss with all patients is important—any weight loss will help, 10% better than 5% [18].

Use of motivational interviewing techniques can be helpful, focusing on gains and goals for the person. There is now increasing evidence for this in OA, with a recent thought-provoking call to action [33] and the emergence of other toolkits supporting positive consultations that empower people with OA [34]. Studies have recently reported on the development and feasibility of mobile applications specifically developed for people with hand OA. These results suggest such applications can serve as a viable and feasible mode of information and treatment delivery [35]. However, further research including high-quality trials evaluating the efficacy and cost-effectiveness of this delivery mode is now needed.

## Exercise, splinting and activity modification

### Exercise

EULAR recommends a regimen of range of motion and strengthening exercises [8]. Several recent high-quality trials illustrate a consensus expert opinion–derived hand strengthening and stretching program that informs clinical practice. Dziedzic *et al*. [36] and Kjeken *et al*. [37] both reported stretching and strengthening exercises for osteoarthritic fingers, thumbs and wrists, whereas the OTTER II exercise program [38] is specific to OA at the base of the thumb. The OTTER II program (Table 4) provides accessible patient-oriented OA education and a progressive exercise program. While a 2017 Cochrane systemic review reported low-quality evidence for small to moderate beneficial effects of exercise on hand pain, function and stiffness [39], we nonetheless strongly advocate hand exercise for patients. Exercise may improve pain and stiffness in the very short term, i.e. for a number of hours, and thereby improve function; to our knowledge, the short-term effectiveness of hand exercise has not been investigated.

Uncertainty exists regarding the most effective mode and corresponding level of supervision for hand exercises, whether this be one on one, an exercise group or home-based independent exercise. Some evidence suggests that exercising for 20 min three times a week with rest days in between is a well-tolerated and effective exercise dose [37, 38]. Allowing recovery days between sessions tries to avoid increases in pain or inflammation, promoting adherence. In our clinical practice, we frequently recommend daily exercises broken down into shorter exercise periods across two or three sessions, which seems to improve long-term adherence without exacerbating symptoms.

**Figure 1.**
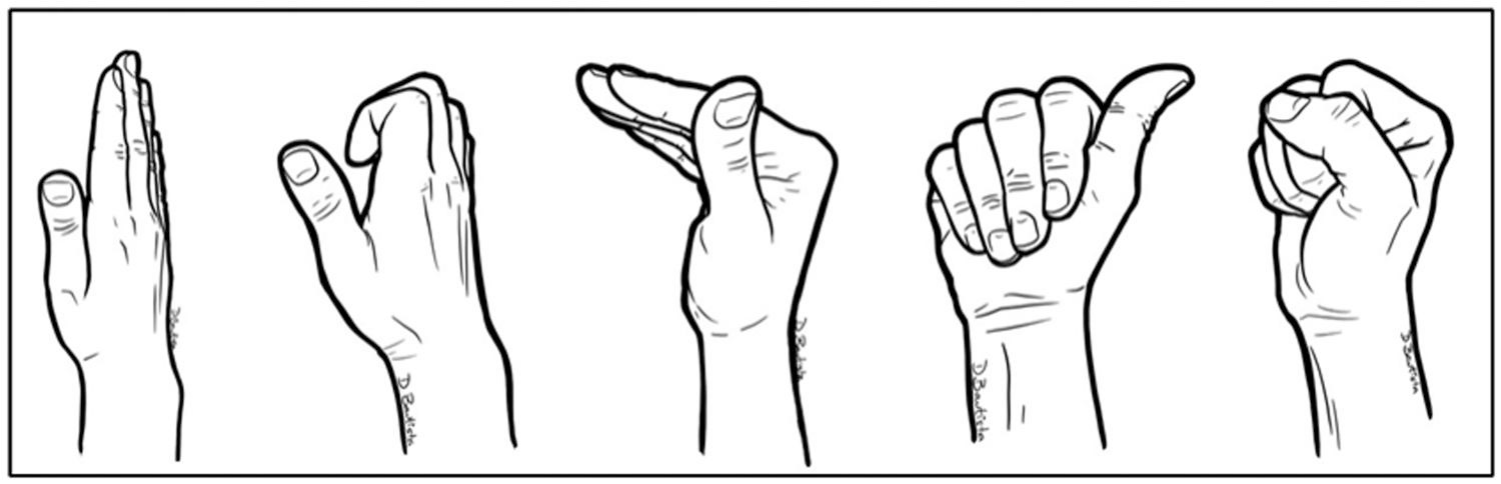
Tendon gliding exercises for hand OA. The five serial tendon gliding exercise positions stretch the long flexor tendons and the intrinsic muscles and encourage stretching of the IP joints into the end available range of motion. Figure used with permission from Daniel Bautista

Patients with hand function deficits are common and are routinely (in our clinical practice) referred to hand therapy for evaluation, education and exercise instruction, aiming to develop their knowledge and skills in symptom self-management. Exercise advice is informed by the OTTER II program [38] with modification based on any identified deformity. For IP joint OA, we additionally routinely use tendon gliding exercises (Fig. 1), with additional thumb mobility and hand strengthening exercises as required [37].

### Splinting

**Figure 2.**
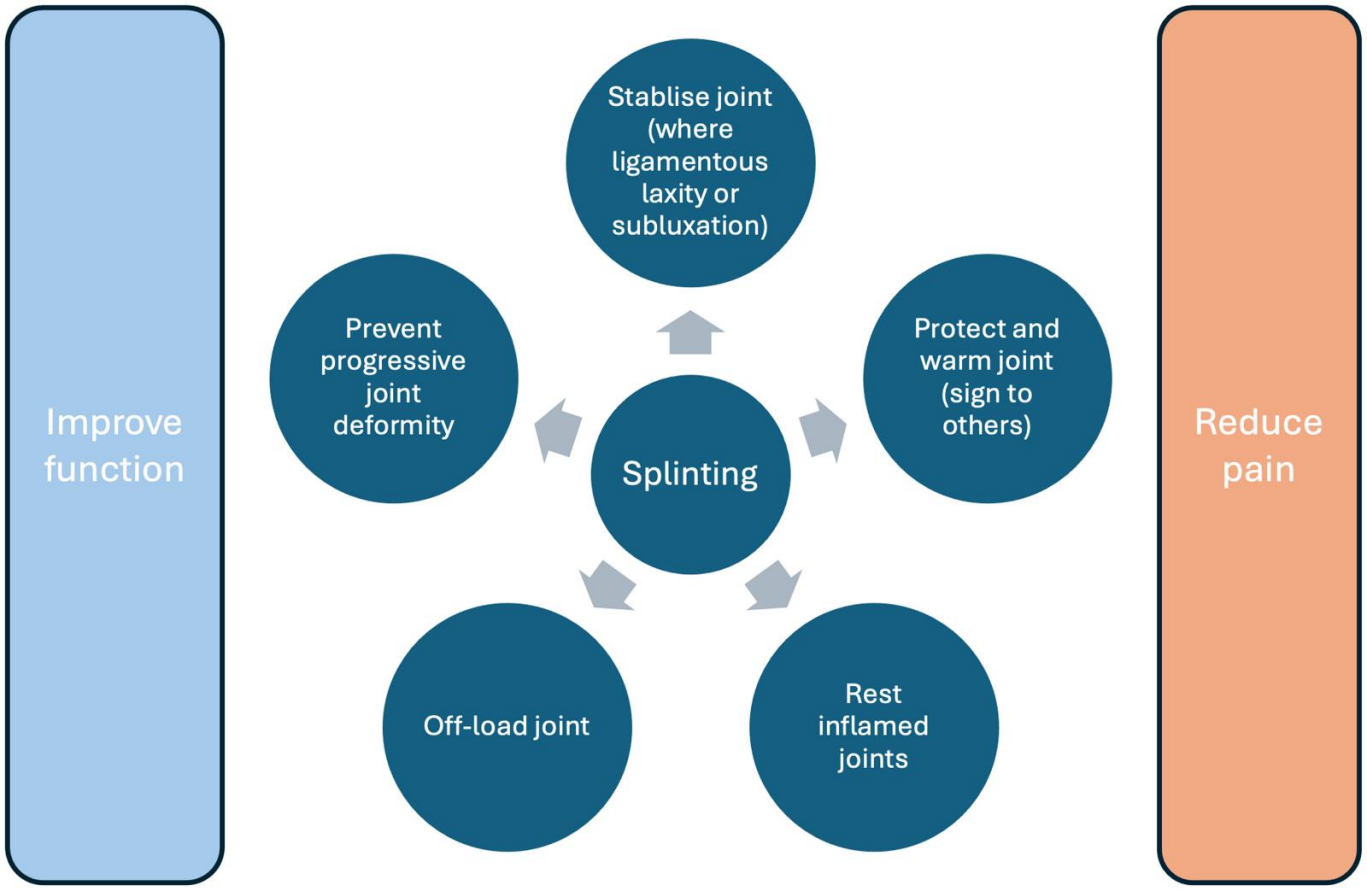
Putative mechanisms and modes of action of hand splints. A variety of mechanisms have been proposed for splinting, most of which are not proven. Their effects are likely to be multifactorial. These may vary in terms of their effects in different clinical scenarios and based on the type of orthotic and how and when they are used. All splints aim to reduce pain, improve function and may prevent or correct joint deformity

**Figure 3.**
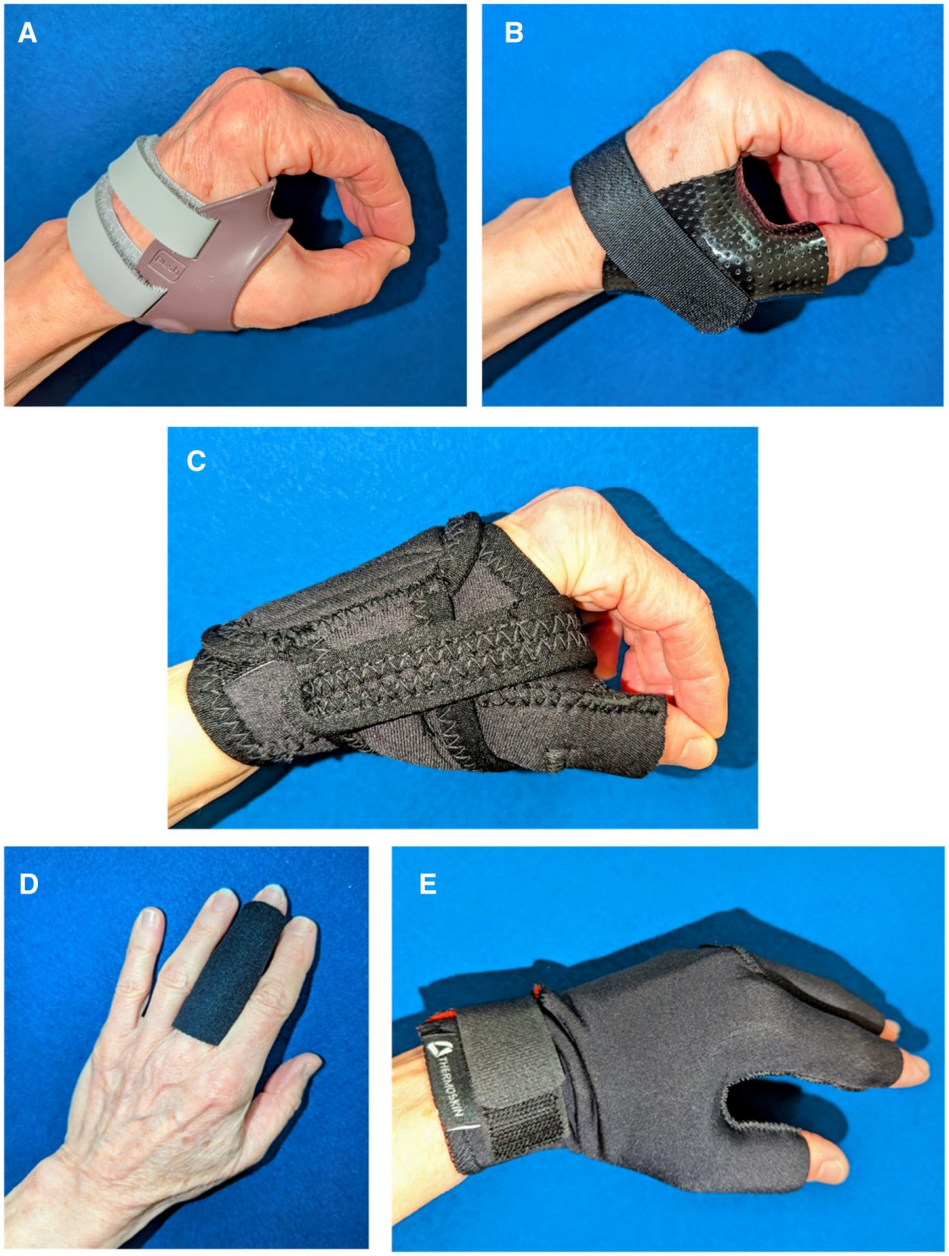
Examples of orthoses for hand OA. **(A–C)** Thumb basal joint OA orthoses. (A) Push thumb brace (example: Nea International bv, Maastricht, The Netherlands). Consider for those with high-load hands who experience pain during function; best for those without a significant positive shoulder sign and with a stable metacarpophalangeal (MCP) joint. (B) Custom thermoplastic splint. For those with pain during function, subluxation of the basal joint and hyperextension deformity of the MCP joint. (C) Ventilated thumb restriction splint (example: Promedics, London, UK). Can be worn for function and at rest, provides warmth and compression. Straps support the basal joint during pinch while encouraging span of the thumb web. Does not stabilize hyperextension deformity of the MCP joint. **(D, E)** Compression garments for hand OA. (D) Compression sleeve for painful IP joints. Custom fabricated or commercially available. Rigidity of material provides support to lateral stress. (E) Thermal compression gloves (example: Thermoskin, Sea-Band Ltd, Leicestershire, UK). Provides warmth and compression across the hand. Fingertips are free for function

The rationale for splinting of the osteoarthritic base of the thumb joint is that support or counterpressure at the CMC joint during functional resistive pinch activities may address various issues (Fig. 2). Their use for these reasons has been recently supported by a consensus group as part of evidence-based British Society for Surgery of the Hand Evidence for Surgical Treatment (BEST) guidelines (Table 1). There are numerous commercially available splints (orthoses) for support or protection of the thumb basal joint (Fig. 3A–C). Additionally, custom orthoses in various designs can be fabricated by clinicians with the requisite skills, using low-temperature thermoplastics. Prefabricated orthoses may be preferred by patients; a custom orthosis is suggested when firmer support or additional stability is desired to improve thumb posture.

However, the trials evidence for effectiveness of splinting is equivocal. The OTTER II trial found splinting at the base of the thumb provided no additional benefit to a supported self-management program of education and exercise delivered by therapists [40]. These were trial-specific orthoses [38], worn over a period of 8 weeks, and findings may not be generalizable to all thumb orthoses or intervention periods.

There has been considerably less research investigating the effectiveness of splinting for osteoarthritic IP joints. A 2019 systematic review concluded that splinting of DIP joints is effective in reducing pain [41]. We investigated night-time custom gutter thermoplastic DIP joint splinting over 3 months in a controlled trial, showing joint pain reduction and improvement in joint extension [42]. Night-time use aimed to avoid substantial loss of joint flexion, and indeed this did not occur. Another study of a bespoke manufactured thermoplastic splint design worn all day similarly identified a reduction in pain [43].

**Figure 4.**
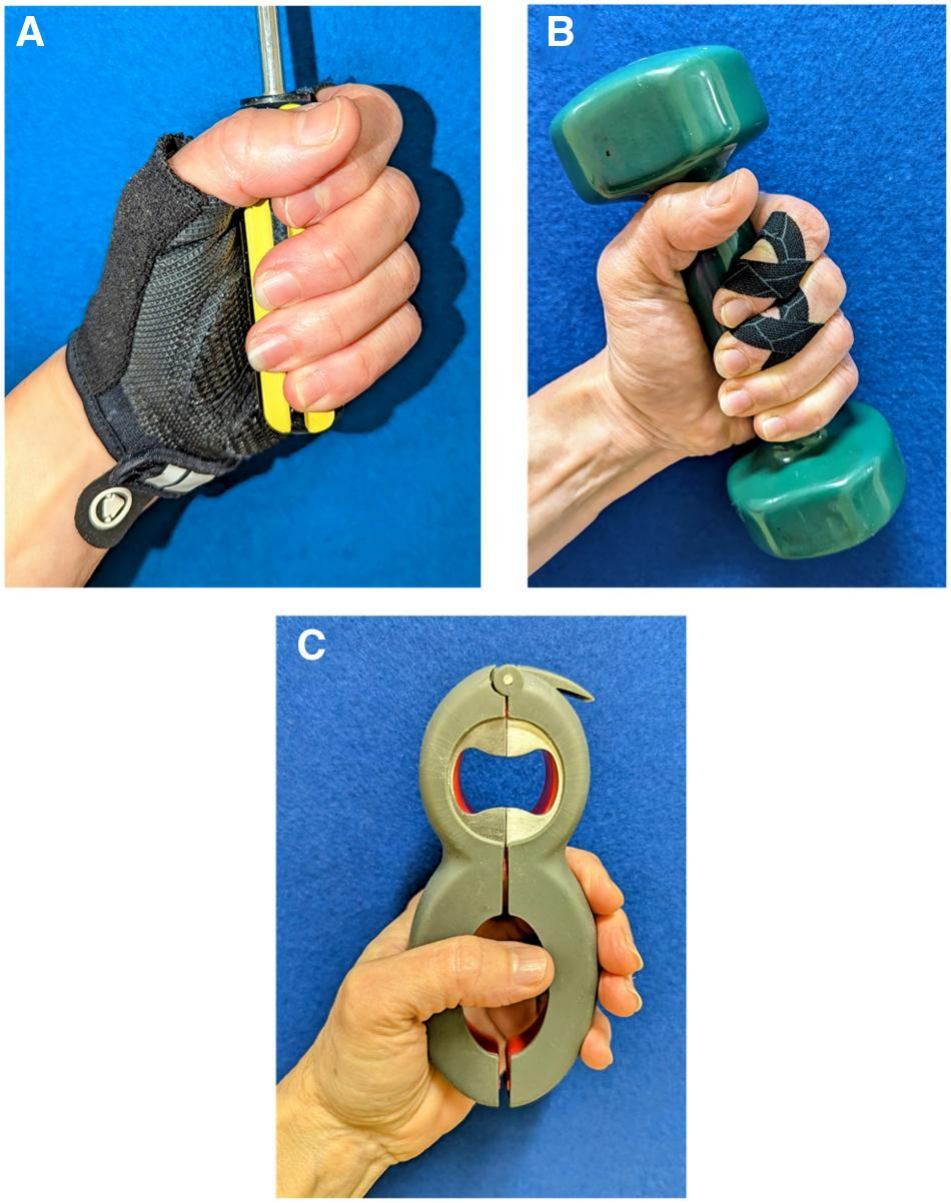
Activity modification and joint offloading for hand OA. **(A)** Cycling gloves (example: Endura mitt, Endura, Cheshire, UK). Padding protects the median nerve at the carpal tunnel and Al pulleys at the metacarpal heads. A textured palm facilitates grip. Joint protection like this is recommended during heavy household tasks, gardening and sports. **(B)** Figure-eight taping. Taping may reduce lateral stress on the DIP joints and reduce pain during heavy functional activity and sport without loss of joint mobility. **(C)** Adaptive devices (example: 6-in-1 multi-opener, Progressive, Kent, WA, USA). Reduces load on thumb and finger joints during domestic and work tasks. Demonstration video available from https://www.youtube.com/watch? v=zuKEq5F1974

There is scant evidence regarding splinting of PIP joints. In our clinical practice, we are reticent to immobilize them, as any loss of active motion can have profoundly negative consequences for hand function. As opposed to static orthoses, we consider the use of light compression garments made of soft stretch fabric (individual finger sleeve or whole hand) that, though lacking an evidence base for hand OA, aim to support pain relief and enhanced function (Fig. 3D and E). Compression could reduce inflammation, reduce pain by diverting attention or stimulate large afferent nerve fibres (gate control theory). In our clinical experience, patients with base of the thumb and IP joint pain report relief with such intermittent light compression. They may purchase these themselves to try if they are not available by healthcare routes. Taping (such as seen in Fig. 4B) may also reduce lateral stress and improve joint pain.

### Activity modification and joint protection

Education in the principles of joint care (joint protection) is important to encourage offloading joints during functional tasks (Table 4). This is not advice to avoid the use of joints, but to help reduce strain on joints during all activities of daily living. Greater force is required when gripping smaller diameter objects—cushioned gloves can be helpful (Fig. 4A) or patients may find that building up tool handles is helpful. Adaptive devices can also be useful for offloading the joints (Fig. 4C).

## Pharmacological options

According to NICE guidance, drugs should not be used as first-line treatment, which is sometimes unsettling to specialist musculoskeletal services that are increasingly focused on drug treatment for other arthropathies [7]. That being said, by the time people reach secondary care, a raft of measures may be useful in controlling symptoms, with drugs being one of them.

### NSAIDs

Topical NSAIDs are a recommended first-line pharmacological treatment for hand OA with proven efficacy, but often underused [7]. They are particularly helpful for one or a few affected joints, especially the base of the thumb or DIP joints. Many patients are unaware of this recommendation or how to use them. Gels tend to be more soothing than creams. It is best to apply sparingly once or twice daily—starting at night may be less inconvenient. Patients should wash their fingers after use. Cotton gloves may be preferred.

NICE does not recommend oral anti-inflammatories [NSAIDs or cyclooxygenase-2 (COX-2) inhibitors] as a first-line option for OA, but they may be considered when other treatments are ineffective or not tolerated [7]. They are only used by ≈15–25% of OA patients, many of whom have contraindications [44]. Clinicians should follow guidelines to use the lowest dose for the shortest duration and counsel and review use regularly, including cardiovascular and gastrointestinal risks in longer-term users. NSAIDs can help manage flares. For long-term use (>2 weeks), patients should take them with meals and a proton pump inhibitor [45]. While long-term monitoring guidance is lacking, we would also recommend annual checks for blood count, liver and renal function and blood pressure, with more frequent monitoring if needed. This applies to both conventional NSAIDs and COX-2 inhibitors.

### Other analgesics

Opiates are not routinely recommended for treatment of painful hand OA [7]. Paracetamol (acetaminophen) was also removed from NICE guidance, as its overall efficacy in trials is low [46]. This evidence tends to come from trials of single large-joint OA. In our opinion, it still offers a less toxic alternative to oral anti-inflammatories, which we advocate as an option where it is found helpful, based on the need for an analgesic ladder for this chronic painful condition.

### Steroids

The use of injections for hand OA remains controversial [47–49]. Trials of injections at the base of the thumb show a short-lived effect (4–6 weeks), potentially driven at least in part by placebo, regression to the mean or contextual factors. There is less evidence for or against PIP joint injections [48]. The EULAR guidance questions the continued use of injections at the base of the thumb [8]. However, given the limited pharmacological and surgical options, injections are still considered for severe pain or flare and may reduce longer-term use of other analgesics [50]. Concerns exist about the long-term effects of cumulative steroid injections or local anaesthetics, as seen in knee OA [51]. Practical considerations for hand joint injections are summarized in Table 5.

**Table 5.** Practical suggestions when considering intra-articular steroid injections in hand joints

Consideration	Consider	Avoid
Timing	Consider only where other interventions (including non-drug) have failed; where there is flare or persistent moderate to severe pain/synovial swelling; where the patient is comfortable with the indication (for symptoms) and associated risks	Use first-line before other strategies (non-drug and drug) have been tried Do not advocate to the many patients who do not want injections—they can usually be managed by other means
Agent	We would tend to use a long-acting steroid (such as methylprednisolone acetate), although soluble steroids can also be used	Combine steroid with large amounts of local anaesthetic—use of 1% lidocaine at <20% of total volume, or none is advisable
Volume	A total injection volume of 1 ml is acceptable for first CMC injections Lower volumes (i.e. ≥0.5 ml) are better tolerated for the IP joints	Use high volumes or inject under pressure if there is a large effusion—they can cause significant capsular pain and risk of ‘leaching back’ with associated subcutaneous tissue atrophy We would advise against injecting the DIP joints: this is particularly painful and anecdotally joint fusion has been reported
Frequency	Make clear that this is a single injection, that courses are not advocated If effective but wearing off, other interventions may be considered	Avoid closely spaced injections to the same joint (<3 month interval) Avoid multiple injections (e.g. >3 to one joint) wherever possible (crystalline matter in these preparations on repeated use appears to cause a paradoxical synovitis in some)
Advice	Combine with splinting or prescribed rest for a few days (including time off work where relevant) Active mobilization should be encouraged from 1 week after injection to improve joint range of motion and reduce the risk of joint fusion, particularly for IP joints	Post-injection pain in the 1–2 days following injection is not uncommon and should be managed with paracetamol (with advice/vigilance for features of joint infection)

Trials of oral and intramuscular steroids have shown pain relief, although doses of 10 mg/day are needed and rebound pain occurs after stopping [52, 53]. Long-term steroid use is not recommended due to the established risks [54], but, as in other rheumatology practice, short courses or single intramuscular doses may sometimes help gauge whether a patient could benefit from off-label immunosuppressants like methotrexate.

### Diet and nutraceuticals

Advice should be given to maintain a healthy, balanced diet and use multivitamins if needed, along with vitamin D. There is no evidence that vitamin D levels (or its supplementation) have therapeutic effects on OA and high-quality evidence is lacking for other nutraceuticals [55, 56]. Chondroitin sulphate has been studied for hand OA [57]. Response to other popular remedies such as turmeric, curcumin or special diets could be due to placebo or contextual effects. These agents are usually combined with proven treatments like hand exercises or weight loss. While most supplements are safe, they can be costly, so in our opinion care should be taken in recommending them. If patients choose to use them, they should be encouraged not to start at a time when they are changing other things and monitor their symptoms for 3–6 months before deciding whether to continue.

### Other pharmacological agents

Topical capsaicin (0.025–0.05%) can be helpful for chronic pain in hand OA, especially with neuropathic features over the base of the thumb, but should be used sparingly with caution highlighted, as it can irritate the eyes and sensitive areas. Duloxetine may be beneficial for hand OA associated with sleep disturbance or anxiety, particularly if there is another indication for a selective serotonin reuptake inhibitor [58]. Oral colchicine trials for hand OA have been negative, but it may be useful if secondary crystal diseases like calcium pyrophosphate deposition disease (CPPD) or hyperuricaemia are suspected (see below).

There are no licensed disease-modifying therapies for OA (see Emerging treatments). Hydroxychloroquine is not effective and should not be used unless another coexisting disease is being treated [59]. Other biologic agents blocking cytokines, such as anti-TNF, IL-1 inhibition or IL-6 inhibition, should not be used, based on trial evidence of a lack of efficacy in hand OA [54, 60–63].

## Surgical options

Hand surgery is usually reserved for symptomatic disease that has exhausted non-surgical treatments, but in our experience it is rarely considered by the rheumatologist. Patients considering surgery often have tried topical and oral analgesia, hand therapy, with or without splinting, and steroid injections. Surgical management of hand OA involves a spectrum of procedures tailored to the severity of symptoms, degree of functional impairment and patient-specific factors, such as activity demands. The main procedures include joint denervation, arthrodesis and arthroplasty.

### DIP joint

The preferred surgical option is arthrodesis (joint fusion). While implant arthroplasty is possible, it may result in joint instability while preserving little movement. Arthrodesis provides reliable pain relief and a stable and dependable fingertip with correction of any deformity. The procedure can be performed under local anaesthetic. Many arthrodesis techniques are described, each with their own indications [64].

### PIP joint

Both arthrodesis and implant arthroplasty are considered for the PIP joint [65]. The choice is dependent on which finger is involved and patient preference. Patients with stiff immobile joints will often chose arthrodesis. Implant arthroplasty is usually achieved with a Swanson silicone spacer [66]. Surface replacement implants are available but are more expensive and associated with higher revision rates.

### Base of the thumb

Trapeziectomy (also known as trapeziumectomy) was first described in 1949, in which the trapezium bone is removed to treat trapeziometacarpal joint OA. There have been >30 modifications or other procedures described since then. The current NICE-accredited surgical guidelines, published by the BSSH, recommend trapeziectomy alone as the preferred surgical option (Table 1). Trapeziectomy can be augmented with ligament reconstruction and tendon interposition. The additional surgical complexity does not appear to improve outcomes and may increase the risk of harm [67]. Implant arthroplasty rates have increased over the last 5 years but currently lack high-quality evidence to support their use [68]. Implants may shorten recovery time but might not improve long-term outcomes and are more costly.

## Other considerations in the management of hand OA

### Therapeutic heat modalities

Many patients report temporary pain relief and improved hand mobility after using therapeutic heat, although evidence for effectiveness in hand OA is weak [69]. Heat modalities, such as hot packs, paraffin wax baths, electric mittens or simply immersing hands in warm (not hot) water, are believed to increase blood flow, metabolism and connective tissue elasticity, theoretically improving function and reducing pain [70]. However, caution is needed to avoid burns, especially in patients with reduced sensation from conditions like diabetes mellitus (DM) or carpal tunnel syndrome (CTS). Hand therapists often offer a trial of paraffin wax therapy to help patients make informed decisions before purchasing a bath for home use. While heat (including before doing hand exercises) can support symptom self-management, its routine use in clinical care lacks strong evidence.

### Flares

Inflammatory flares are common in hand OA, but gout or Calcium pyrophosphate deposition disease (CPPD) should be ruled out. In cases of hyperuricaemia, treatment is recommended even without a clear gout diagnosis. For inflammatory joint symptoms with frequent, short-lived flares suggestive of CPPD, a short course of colchicine can be tried (monitoring clinical response). If hyperuricaemia is present, allopurinol should be used to suppress urate levels to <360 μmol/l, as per NICE guidance [71].

In our experience, offloading is likely to help hand symptoms, reducing the frequency of flare episodes (with offloading further helping to ameliorate flares when they occur). Consider occupational, home and exercise activities that can be modified to bring about offloading as well as encouraging the use of orthoses.

### Sleep

NICE guidance does not include that sleep hygiene is important for those with OA. Even in the general population, there is good evidence that impaired sleep is associated with small elevations in inflammatory markers and a decline in self-reported physical health status [72]. Poor sleep and night pain are not uncommon in hand OA and may potentiate pain syndrome, particularly in post-menopausal women. Asking about this is often helpful, and general advice and signposting should be given.

### Imaging

The question of when to consider imaging often arises. While X-rays are not required for diagnosis, they can help guide treatment decisions, such as splinting, injection or surgery. In cases of diagnostic uncertainty, X-rays can detect periosteal changes in seronegative inflammatory arthritis or chondrocalcinosis from CPPD. Ultrasound is a useful option for assessing atypical features and quantifying synovitis. MRI is rarely needed unless other conditions are being considered or for trial purposes, as an attempted stratification method (discussed in Marshall *et al.* [2] and Favero *et al.* [17] and also ‘The opportunity and clinical need for stratification’ below) or outcome measure; a more detailed exploration of imaging is outside the scope of this review.

### Common concurrent/associated common musculoskeletal conditions of the hand

Flexor tendinopathy and CTS are common in hand OA patients, sharing risk factors or being influenced by the underlying disease. It is important to detect and manage these treatable conditions, which can cause digital, hand or thumb base pain, reducing hand function further. It should be noted that OA-related remodelling and sensitivity at the base of the thumb may affect the fit and tolerance of prefabricated resting splints for CTS, which sometimes requires custom orthoses.

### Comorbidities and metabolic syndrome

Metabolic syndrome, often undiagnosed, is prevalent in this patient group, including hyperlipidaemia and type 2 DM [19, 32]. There is active investigation into whether these comorbidities have shared risks or have a bearing on the disease course [73–75]. Ensuring active detection and management as we would in inflammatory arthritis or vasculitis appears important. Lupus, fibromyalgia and polymyalgia rheumatica were also found to be more likely in OA populations [74].

## Future and emerging treatments relevant to practice

### Existing anti-rheumatic agents

Two international trials of oral methotrexate for hand OA have shown opposing results, though the negative trial only used a dose of 10 mg/week [54, 76, 77]. The other, the METHODS trial, met its primary endpoint of pain reduction over 6 months, using 20 mg of methotrexate per week [76]. Here, an attempt at stratification at inclusion of those with some level of targetable inflammation meant participants required MRI-detected grade 1 synovitis or more for enrolment. It is not yet clear what bearing this MRI criterion has for generalizability of findings to the clinic, where patients would not typically undergo MRI, including for treatment decisions. It will be important to understand if there are reasonably reliable clinical or ultrasound-based correlates, or even whether such stratification is necessary at all.

Methotrexate should not yet be routinely used for hand OA (based on one trial), but could be considered in exceptional cases with a dosing regimen similar to that for RA (15–20 mg/week with folate supplementation). In our clinical experience, this should be done only after consultation with at least one colleague and/or in a multidisciplinary team meeting, when objective features suggest severe disease, diagnostic uncertainty/possible overlap with seronegative arthritis, progressive radiologically erosive polyarthropathy, uncontrolled hand pain (≥6/10), active synovitis in two or more joints and/or low-grade C-reactive protein (CRP) response. The patient should agree to off-label use, outside of NICE guidance, and provide informed consent. Baseline and follow-up measurements (e.g. pain, function, joint counts) should be recorded and therapy should be stopped if there is no response after adequate dosing. Prescreening and monitoring should follow RA guidelines.

To our knowledge there are no clinical trials examining sulfasalazine in hand OA, although there are some case series data. Anecdotally patients who show overlap features with seronegative arthropathy may benefit, although caution should be exercised given that clinical trial evidence is lacking.

### Bone remodelling drugs

One clinical trial of denosumab in those with erosive hand OA showed some slowing of structural deterioration, with an impact on pain described in the extension phase [78]. There is no evidence for efficacy of bisphosphonate therapy in hand OA [79]. At present, neither class is recommended for treating hand OA.

### HRT

There has been interest in the link between menopause, hormone loss and hand OA, particularly in peri- or postmenopausal women [80–82]. Currently there is no specific recommendation beyond standard guidance for using HRT in those with painful hand OA. However, a history of menopause timing, hormone use and related symptoms can help determine whether heightened hand pain might be part of a broader menopausal syndrome. Musculoskeletal pain (myalgia, arthralgia) is a common menopausal symptom that may be considered when initiating HRT under current NICE guidance [83]. HRT should not be abruptly stopped unless for safety reasons, as tapering over 4 weeks can cause a flare in hand OA symptoms [84]. Although understanding is incomplete, findings suggest that a decrease in sex hormones, especially when rapid, may play a role in hand OA for some women [85].

### Glucagon-like peptide-1 (GLP-1) agonists

Like many branches of medicine, we are trying to fully understand the role and place in the management in OA beyond current licenses of GLP-1 agonists. So far, trials have been focused on the knee and have shown pain reduction [86]. There is some evidence they may reduce inflammation in the joints and alleviate pain by targeting the inflammatory pathways, which may be relevant for the condition [87]. Further hand-specific data on the effects of this drug class will be of great interest.

### New pharmacological agents in trials

New or repurposed drug classes are being tested that are linked to pathogenic mechanisms arising from GWASs. For example, drugs that boost levels of retinoic acid and suppress joint degradation in preclinical models [30] are currently being tested in human hand OA. Other genetic associations with hand OA have recently been described and may support new drug targets or tests that will help to subgroup this disease [28, 88]. This is important, as it appears that having a genetic association for any given pathway more than doubles the chances of a successful therapeutic being developed [89].

### Extending the evidence base for surgical intervention

While high-quality randomized controlled trials have compared surgical techniques, no studies have compared surgery with conservative treatment. The Surgery versus Conservative OsteOarthritis of Thumb Trial (SCOOTT) has recently been funded. The aim of the study is to answer two questions: Is surgery, trapeziectomy or carpometacarpal joint replacement (CMCJR) superior to enhanced non-surgical management? and Is CMCJR non-inferior to trapeziectomy? Another study, the Finnish Thumb Arthritis Surgery Trial (FINTASY) is investigating the efficacy of trapeziectomy with placebo surgery as the comparator. Surgical techniques also continue to evolve. Surgical joint distraction preserves the native joint and appears effective for OA at other sites, but the benefits might not outweigh the inconvenience of the device in the hand [90].

### The opportunity and clinical need for stratification

There have been recent efforts to better stratify patients with hand OA to select those most likely to respond to therapy. This ranges from their inclusion into trials (such as the presence of a more inflammatory phenotype for agents targeting inflammation, e.g. imaging-based presence of synovitis by ultrasound or MRI [2, 76]), or in the assessment of outcomes (e.g. bone change for bone-targeting agents [78]). So far, there is little definitive evidence supporting our ability to identify clinically meaningful, discrete molecular subtypes in hand OA or that we might successfully ‘deep-phenotype’ responders to treatments. However, it remains intuitive that those with a more active or aggressive disease course, or certain disease features relating to the therapeutic target, may better demonstrate response to therapy within a trial period. Progress here may yet improve our success rates in identifying effective interventions.

There are significant gaps in our knowledge and effective management options for hand OA, as highlighted in this review. Recent research priority-setting exercises in this area are summarized, alongside key research gaps, in Table 6 [91, 92]. Research gaps highlighted in the updated NICE recommendations correlate poorly with these [7]. While not the main purpose of this review, research could better represent the voice of the patient and mobilize knowledge into current pathways of care. There is an apparent and increasing inequity in managing OA, partly due to perceived prevalence (inevitability/relation to ageing) and relative importance (and associated tolerization). This is true for rheumatology and likely in other healthcare areas, including primary care. As clinicians, we question whether we are doing enough for those affected by common musculoskeletal conditions such as hand OA.

**Table 6.** Research gaps, opportunities and barriers for hand OA.

Research need	Research setting process and or reference where relevant	Opportunity	Barrier
New interventions that either target pain or slow or prevent disease are needed. These should be targeted to specific pathogenic mechanisms	Child Health and Nutrition Research Initiative (CHNRI) method; common musculoskeletal conditions including osteoarthritis [91]	Quality of life; primary care consultations; reduce need for surgical procedures	Efficacy in current trials
When interventions should be used and in whom Subgrouping that is useful clinically and in trials of those with hand OA to allow stratification of populations or their selective inclusion into trials is needed, ideally with molecular tests that do this	James Lind Alliance Priority Setting Process (PSP) in common conditions of hand and wrist [92]	Would support better care pathways and equity for patients Would make new treatments more cost effective	Lack of relevant clinical subgroups or phenotyping Lack of stratification Lack of prognostic biomarkers
Better trial design		Needed in OA as a whole	Range of issues with definitions of included population and sensitivity and specificity of outcomes
Meaningful clinical outcomes Documentation and measurement of flare		May include periods of flare or symptoms important to patients as well as pain	Assessment of pain at static time points does not take into account disease variability
Diagnostic coding of cases as ‘hand osteoarthritis’ (irrespective of stage or subgroup) in electronic health records		Advisable for homogeneity and the disease being better ‘counted’ (and considered) by healthcare systems	Critical barrier for both healthcare provision, electronic health records–based research and participant identification for studies/trials. OA is often undercoded (non-specific codes such as ‘hand pain’ or ‘arthritis NOS’ being frequent)
Core standardized pain and functional assessments	Outcome Measures in Rheumatology (OMERACT) hand OA working group (who are carrying out ongoing work in this area) [93]	Routinely collected data that cross over to trials (much like DAS28 in RA) would allow better patient care, knowledge transfer and opportunity for research with electronic health records data	No standardized outcomes for people with OA in general or hand OA
Reasons for barriers to care		Effective management, supported by good communication about the best evidence on the condition	Barriers to access to clinical care, high-quality information and treatment exist and may include: lack of diagnosis; lack of multidisciplinary team input, including access to therapies; subconscious or other biases relating to lower clinical priority for common musculoskeletal conditions; lower educational/socio-economic status, cultural or language issues reducing health advocacy and healthcare access; gender-based referral and treatment biases may exist around painful conditions in females

## Conclusions

Hand OA remains the Cinderella of the rheumatology clinic. While biologic or targeted therapies are lacking, evidence-based interventions and positive consultation approaches can greatly improve the lives of those affected. This review aims to provide a practical toolkit for rheumatologists and others to better support patients with this common yet often misunderstood condition.

## Data Availability

No new data were generated or analysed in support of this article.

## References

[1] Versus Arthritis. The state of musculoskeletal health 2021: arthritis and other musculoskeletal conditions in numbers. London: Versus Arthritis, 2021.

[2] Marshall M , Watt FE, Vincent TL, Dziedzic K. Hand osteoarthritis: clinical phenotypes, molecular mechanisms and disease management. Nat Rev Rheumatol 2018;14:641–56. 10.1038/s41584-018-0095-4 30305701

[3] Peat G , Rathod-Mistry T, Paskins Z et al Relative prevalence and distribution of knee, hand and foot symptomatic osteoarthritis subtypes in an English population. Musculoskelet Care 2020;18:219–24. 10.1002/msc.1457 31995282

[4] Slatkowsky-Christensen B , Mowinckel P, Loge JH, Kvien TK. Health-related quality of life in women with symptomatic hand osteoarthritis: a comparison with rheumatoid arthritis patients, healthy controls, and normative data. Arthritis Rheum 2007;57:1404–9. 18050180 10.1002/art.23079

[5] Punzi L , Ramonda R, Sfriso P. Erosive osteoarthritis. Best Pract Res Clin Rheumatol 2004;18:739–58. 10.1016/j.berh.2004.05.010 15454130

[6] Gulati M , Brewer G, Judge A et al Could sex-specific subtypes of hand osteoarthritis exist? A retrospective study in women presenting to secondary care. Front Pain Res (Lausanne) 2024;5:1331187. 38410176 10.3389/fpain.2024.1331187 PMC10895010

[7] National Institute for Health and Care Excellence. Osteoarthritis in over 16s: diagnosis and management. NG226. London: National Institute for Health and Care Excellence, 2022. 36745715

[8] Kloppenburg M , Kroon FP, Blanco FJ et al 2018 update of the EULAR recommendations for the management of hand osteoarthritis. Ann Rheum Dis 2019;78:16–24. 30154087 10.1136/annrheumdis-2018-213826

[9] Bannuru RR , Osani MC, Vaysbrot EE et al OARSI guidelines for the non-surgical management of knee, hip, and polyarticular osteoarthritis. Osteoarthritis Cartilage 2019;27:1578–89. 10.1016/j.joca.2019.06.011 31278997

[10] Kolasinski SL , Neogi T, Hochberg MC et al 2019 American College of Rheumatology/Arthritis Foundation guideline for the management of osteoarthritis of the hand, hip, and knee. Arthritis Rheumatol 2020;72:220–33. 31908163 10.1002/art.41142 PMC10518852

[11] Gangopadhyay S , Jansen V, Sim Khor W et al Guideline on managing thumb base osteoarthritis: the British Society for Surgery of the Hand Evidence for Surgical Treatment (BEST) findings and recommendations. J Hand Surg Eur Vol 2025;50:576–86. 10.1177/17531934241313206 39852312

[12] Bordvik DH , Prior Y, Bamford R et al Development of quality indicators for hand osteoarthritis care—results from an European consensus study. Osteoarthr Cartil Open 2025;7:100578. 39995586 10.1016/j.ocarto.2025.100578 PMC11849605

[13] Verbruggen G , Veys EM. Numerical scoring systems for the anatomic evolution of osteoarthritis of the finger joints. Arthritis Rheum 1996;39:308–20. 8849385 10.1002/art.1780390221

[14] Keen HI , Wakefield RJ, Grainger AJ et al An ultrasonographic study of osteoarthritis of the hand: synovitis and its relationship to structural pathology and symptoms. Arthritis Rheum 2008;59:1756–63. 19035429 10.1002/art.24312

[15] Tan AL , Grainger AJ, Tanner SF, Emery P, McGonagle D. A high-resolution magnetic resonance imaging study of distal interphalangeal joint arthropathy in psoriatic arthritis and osteoarthritis: are they the same? Arthritis Rheum 2006;54:1328–33. 16575858 10.1002/art.21736

[16] Marshall M , Nicholls E, Kwok WY et al Erosive osteoarthritis: a more severe form of radiographic hand osteoarthritis rather than a distinct entity? Ann Rheum Dis 2015;74:136–41. 24095935 10.1136/annrheumdis-2013-203948 PMC4283656

[17] Favero M , Belluzzi E, Ortolan A et al Erosive hand osteoarthritis: latest findings and outlook. Nat Rev Rheumatol 2022;18:171–83. 35105980 10.1038/s41584-021-00747-3

[18] Messier SP , Mihalko SL, Legault C et al Effects of intensive diet and exercise on knee joint loads, inflammation, and clinical outcomes among overweight and obese adults with knee osteoarthritis: the IDEA randomized clinical trial. JAMA 2013;310:1263–73. 10.1001/jama.2013.277669 PMC4450354 24065013

[19] Courties A , Sellam J, Berenbaum F. Metabolic syndrome-associated osteoarthritis. Curr Opin Rheumatol 2017;29:214–22. 10.1097/BOR.0000000000000373 28072592

[20] van der Meulen C , van de Stadt LA, Rosendaal FR, Runhaar J, Kloppenburg M. Determination and characterization of patient subgroups based on pain trajectories in hand osteoarthritis. Rheumatology (Oxford) 2023;62:3035–42. 10.1093/rheumatology/kead017 PMC10473188 36648311

[21] Brandt KD , Dieppe P, Radin EL. Commentary: is it useful to subset “primary” osteoarthritis? A critique based on evidence regarding the etiopathogenesis of osteoarthritis. Semin Arthritis Rheum 2009;39:81–95. 19796724 10.1016/j.semarthrit.2009.06.001

[22] Vincent TL. Mechanoflammation in osteoarthritis pathogenesis. Semin Arthritis Rheum 2019;49(3 Suppl):S36–8. 31779850 10.1016/j.semarthrit.2019.09.018

[23] Vincent TL. Of mice and men: converging on a common molecular understanding of osteoarthritis. Lancet Rheumatol 2020;2:e633–45. 32989436 10.1016/S2665-9913(20)30279-4 PMC7511206

[24] Burleigh A , Chanalaris A, Gardiner MD et al Joint immobilization prevents murine osteoarthritis and reveals the highly mechanosensitive nature of protease expression in vivo. Arthritis Rheum 2012;64:2278–88. 10.1002/art.34420 22307759

[25] Wiegant K , van Roermund PM, Intema F et al Sustained clinical and structural benefit after joint distraction in the treatment of severe knee osteoarthritis. Osteoarthritis Cartilage 2013;21:1660–7. 23954704 10.1016/j.joca.2013.08.006

[26] McNamee KE , Burleigh A, Gompels LL et al Treatment of murine osteoarthritis with TrkAd5 reveals a pivotal role for nerve growth factor in non-inflammatory joint pain. Pain 2010;149:386–92. 10.1016/j.pain.2010.03.002 20350782

[27] Tan AL , Toumi H, Benjamin M et al Combined high-resolution magnetic resonance imaging and histological examination to explore the role of ligaments and tendons in the phenotypic expression of early hand osteoarthritis. Ann Rheum Dis 2006;65:1267–72. 10.1136/ard.2005.050112 PMC1798321 16627540

[28] Boer CG , Hatzikotoulas K, Southam L et al Deciphering osteoarthritis genetics across 826,690 individuals from 9 populations. Cell 2021;184:6003–5. 34822786 10.1016/j.cell.2021.11.003 PMC8658458

[29] Styrkarsdottir U , Thorleifsson G, Helgadottir HT et al Severe osteoarthritis of the hand associates with common variants within the *ALDH1A2* gene and with rare variants at 1p31. Nat Genet 2014;46:498–502. 24728293 10.1038/ng.2957

[30] Zhu L , Kamalathevan P, Koneva LA et al Variants in *ALDH1A2* reveal an anti-inflammatory role for retinoic acid and a new class of disease-modifying drugs in osteoarthritis. Sci Transl Med 2022;14:eabm4054. 36542696 10.1126/scitranslmed.abm4054

[31] Nicholls E , Thomas E, van der Windt DA, Croft PR, Peat G. Pain trajectory groups in persons with, or at high risk of, knee osteoarthritis: findings from the Knee Clinical Assessment Study and the Osteoarthritis Initiative. Osteoarthritis Cartilage 2014;22:2041–50. 25305072 10.1016/j.joca.2014.09.026 PMC4256061

[32] Marshall M , Peat G, Nicholls E et al Metabolic risk factors and the incidence and progression of radiographic hand osteoarthritis: a population-based cohort study. Scand J Rheumatol 2019;48:52–63. 29952684 10.1080/03009742.2018.1459831 PMC6319183

[33] Jinks C , Botto-van Bemden A, Bunzli S et al Changing the narrative on osteoarthritis: a call for global action. Osteoarthritis Cartilage 2024;32:414–20. 38354847 10.1016/j.joca.2024.02.004

[34] Bowden JL , Hunter DJ, Mills K et al The OARSI Joint Effort Initiative: priorities for osteoarthritis management program implementation and research 2024–2028. Osteoarthr Cartil Open 2023;5:100408. 37771392 10.1016/j.ocarto.2023.100408 PMC10522998

[35] Tveter AT , Varsi C, Maarnes MK et al Development of the happy hands self-management app for people with hand osteoarthritis: feasibility study. JMIR Form Res. 2024;8:e59016. 10.2196/59016 PMC11558211 39470716

[36] Dziedzic K , Nicholls E, Hill S et al Self-management approaches for osteoarthritis in the hand: a 2 × 2 factorial randomised trial. Ann Rheum Dis 2015;74:108–18. 24107979 10.1136/annrheumdis-2013-203938 PMC4283664

[37] Kjeken I , Bergsmark K, Haugen IK et al Task shifting in the care for patients with hand osteoarthritis. Protocol for a randomized controlled non-inferiority trial. BMC Musculoskelet Disord 2021;22:194. 33593307 10.1186/s12891-021-04019-9 PMC7888184

[38] Adams J , Barratt P, Arden NK et al The Osteoarthritis Thumb Therapy (OTTER) II Trial: a study protocol for a three-arm multi-centre randomised placebo controlled trial of the clinical effectiveness and efficacy and cost-effectiveness of splints for symptomatic thumb base osteoarthritis. BMJ Open 2019;9:e028342. 10.1136/bmjopen-2018-028342 PMC6830636 31640992

[39] Osteras N , Kjeken I, Smedslund G et al Exercise for hand osteoarthritis: a Cochrane systematic review. J Rheumatol 2017;44:1850–8. 29032354 10.3899/jrheum.170424

[40] Adams J , Barratt P, Rombach I et al The clinical and cost effectiveness of splints for thumb base osteoarthritis: a randomized controlled clinical trial. Rheumatology (Oxford) 2021;60:2862–77. 10.1093/rheumatology/keaa726 33254239

[41] Beasley J , Ward L, Knipper-Fisher K et al Conservative therapeutic interventions for osteoarthritic finger joints: a systematic review. J Hand Ther 2019;32:153–64.e2. 30017415 10.1016/j.jht.2018.01.001

[42] Watt FE , Kennedy DL, Carlisle KE et al Night-time immobilization of the distal interphalangeal joint reduces pain and extension deformity in hand osteoarthritis. Rheumatology (Oxford) 2014;53:1142–9. 24509405 10.1093/rheumatology/ket455 PMC4023558

[43] Ikeda M , Ishii T, Kobayashi Y et al Custom-made splint treatment for osteoarthritis of the distal interphalangeal joints. J Hand Surg Am 2010;35:589–93. 10.1016/j.jhsa.2010.01.012 20353860

[44] Kingsbury SR , Hensor EM, Walsh CA, Hochberg MC, Conaghan PG. How do people with knee osteoarthritis use osteoarthritis pain medications and does this change over time? Data from the Osteoarthritis Initiative. Arthritis Res Ther 2013;15:R106. 24008023 10.1186/ar4286 PMC3978852

[45] Bradley M. Reducing the risk of NSAID related gastrointestinal problems: an update. Drug Ther Bull 2020;58:89–92. 32234727 10.1136/dtb.2019.000072

[46] Richard MJ , Driban JB, McAlindon TE. Pharmaceutical treatment of osteoarthritis. Osteoarthritis Cartilage 2023;31:458–66. 36414224 10.1016/j.joca.2022.11.005

[47] Riley N , Vella-Baldacchino M, Thurley N et al Injection therapy for base of thumb osteoarthritis: a systematic review and meta-analysis. BMJ Open 2019;9:e027507. 10.1136/bmjopen-2018-027507 PMC6747875 31511280

[48] Kroon FPB , Carmona L, Schoones JW, Kloppenburg M. Efficacy and safety of non-pharmacological, pharmacological and surgical treatment for hand osteoarthritis: a systematic literature review informing the 2018 update of the EULAR recommendations for the management of hand osteoarthritis. RMD Open 2018;4:e000734. 10.1136/rmdopen-2018-000734 PMC6203105 30402266

[49] Dossing A , Nielsen SM, Kroon FP et al Comparative effectiveness of pharmacological interventions for hand osteoarthritis: a systematic review and network meta-analysis of randomised trials. RMD Open 2023;9:e003030. 10.1136/rmdopen-2023-003030 PMC10537980 37734873

[50] Hawley S , Prats-Uribe A, Matharu GS et al Effect of intra-articular corticosteroid injections for osteoarthritis on the subsequent use of pain medications: a UK CPRD cohort study. Rheumatology (Oxford) 2025;64:3832–41. 40036958 10.1093/rheumatology/keaf126 PMC12107031

[51] McAlindon TE , LaValley MP, Harvey WF et al Effect of intra-articular triamcinolone vs saline on knee cartilage volume and pain in patients with knee osteoarthritis: a randomized clinical trial. JAMA 2017;317:1967–75. 10.1001/jama.2017.5283 PMC5815012 28510679

[52] Kroon FPB , Kortekaas MC, Boonen A et al Results of a 6-week treatment with 10 mg prednisolone in patients with hand osteoarthritis (HOPE): a double-blind, randomised, placebo-controlled trial. Lancet 2019;394:1993–2001. 31727410 10.1016/S0140-6736(19)32489-4

[53] Keen HI , Wakefield RJ, Hensor EM, Emery P, Conaghan PG. Response of symptoms and synovitis to intra-muscular methylprednisolone in osteoarthritis of the hand: an ultrasonographic study. Rheumatology (Oxford) 2010;49:1093–100. 20219784 10.1093/rheumatology/keq010

[54] Richette P , Latourte A. Hand osteoarthritis: a fresh look. Joint Bone Spine 2024;91:105652. 37797830 10.1016/j.jbspin.2023.105652

[55] Bergink AP , Zillikens MC, Van Leeuwen JP et al 25-Hydroxyvitamin D and osteoarthritis: a meta-analysis including new data. Semin Arthritis Rheum 2016;45:539–46. 10.1016/j.semarthrit.2015.09.010 26522138

[56] Sofat N , Watt FE, Tan AL. Development of medical therapeutics in osteoarthritis: time for action to improve patient care. Rheumatology (Oxford) 2021;60:3487–9. 10.1093/rheumatology/keab263 PMC8328507 33715006

[57] Gabay C , Medinger-Sadowski C, Gascon D, Kolo F, Finckh A. Symptomatic effects of chondroitin 4 and chondroitin 6 sulfate on hand osteoarthritis: a randomized, double-blind, placebo-controlled clinical trial at a single center. Arthritis Rheum 2011;63:3383–91. 10.1002/art.30574 21898340

[58] Sofat N , Harrison A, Russell MD et al The effect of pregabalin or duloxetine on arthritis pain: a clinical and mechanistic study in people with hand osteoarthritis. J Pain Res 2017;10:2437–49. 29066930 10.2147/JPR.S147640 PMC5644551

[59] Kingsbury SR , Tharmanathan P, Keding A et al Hydroxychloroquine effectiveness in reducing symptoms of hand osteoarthritis: a randomized trial. Ann Intern Med 2018;168:385–95. 29459986 10.7326/M17-1430

[60] Kloppenburg M , Ramonda R, Bobacz K et al Etanercept in patients with inflammatory hand osteoarthritis (EHOA): a multicentre, randomised, double-blind, placebo-controlled trial. Ann Rheum Dis 2018;77:1757–64. 30282670 10.1136/annrheumdis-2018-213202

[61] Aitken D , Laslett LL, Pan F et al A randomised double-blind placebo-controlled crossover trial of HUMira (adalimumab) for erosive hand OsteoaRthritis—the HUMOR trial. Osteoarthritis Cartilage 2018;26:880–7. 29499287 10.1016/j.joca.2018.02.899

[62] Kloppenburg M , Peterfy C, Haugen IK et al Phase IIa, placebo-controlled, randomised study of lutikizumab, an anti-interleukin-1α and anti-interleukin-1β dual variable domain immunoglobulin, in patients with erosive hand osteoarthritis. Ann Rheum Dis 2019;78:413–20. 30552176 10.1136/annrheumdis-2018-213336 PMC6390132

[63] Richette P , Latourte A, Sellam J et al Efficacy of tocilizumab in patients with hand osteoarthritis: double blind, randomised, placebo-controlled, multicentre trial. Ann Rheum Dis 2021;80:349–55. 33055078 10.1136/annrheumdis-2020-218547

[64] Dickson DR , Mehta SS, Nuttall D, Ng CY. A systematic review of distal interphalangeal joint arthrodesis. J Hand Microsurg 2014;6:74–84. 25414555 10.1007/s12593-014-0163-1 PMC4235825

[65] Faulkner H , An V, Lawson RD, Graham DJ, Sivakumar BS. Proximal interphalangeal joint arthrodesis techniques: a systematic review. Hand (N Y) 2023;18:74–9. 33682483 10.1177/1558944721998019 PMC9806530

[66] Yamamoto M , Malay S, Fujihara Y, Zhong L, Chung KC. A systematic review of different implants and approaches for proximal interphalangeal joint arthroplasty. Plast Reconstr Surg 2017;139:1139e–51e. 10.1097/PRS.0000000000003260 PMC5407295 28445369

[67] Field J , Buchanan D. To suspend or not to suspend: a randomised single blind trial of simple trapeziectomy versus trapeziectomy and flexor carpi radialis suspension. J Hand Surg Eur 2007;32:462–6. 10.1016/J.JHSB.2007.02.005 17399871

[68] Wajon A , Vinycomb T, Carr E, Edmunds I, Ada L. Surgery for thumb (trapeziometacarpal joint) osteoarthritis. Cochrane Database Syst Rev 2015;2015:CD004631. 25702783 10.1002/14651858.CD004631.pub4 PMC6464627

[69] Valdes K , Marik T. A systematic review of conservative interventions for osteoarthritis of the hand. J Hand Ther 2010;23:334–50; quiz 51. 20615662 10.1016/j.jht.2010.05.001

[70] Nadler SF , Weingand K, Kruse RJ. The physiologic basis and clinical applications of cryotherapy and thermotherapy for the pain practitioner. Pain Physician 2004;7:395–9. 16858479

[71] Neilson J , Bonnon A, Dickson A, Roddy E, Guideline C. Gout: diagnosis and management—summary of NICE guidance. BMJ 2022; 78:o1754. 10.1136/bmj.o1754 36041743

[72] Afolalu EF , Ramlee F, Tang NKY. Effects of sleep changes on pain-related health outcomes in the general population: a systematic review of longitudinal studies with exploratory meta-analysis. Sleep Med Rev 2018;39:82–97. 29056414 10.1016/j.smrv.2017.08.001 PMC5894811

[73] Swain S , Sarmanova A, Coupland C, Doherty M, Zhang W. Comorbidities in osteoarthritis: a systematic review and meta-analysis of observational studies. Arthritis Care Res (Hoboken) 2020;72:991–1000. 31207113 10.1002/acr.24008

[74] Swain S , Coupland C, Mallen C et al Temporal relationship between osteoarthritis and comorbidities: a combined case control and cohort study in the UK primary care setting. Rheumatology (Oxford) 2021;60:4327–39. 33506862 10.1093/rheumatology/keab067 PMC8410005

[75] Watt FE , Wise EM. Osteoarthritis and associated comorbidities: new answers and more questions. Rheumatology (Oxford) 2021;60:3966–8. 33944908 10.1093/rheumatology/keab405

[76] Wang Y , Jones G, Keen HI et al Methotrexate to treat hand osteoarthritis with synovitis (METHODS): an Australian, multisite, parallel-group, double-blind, randomised, placebo-controlled trial. Lancet 2023;402:1764–72. 37839420 10.1016/S0140-6736(23)01572-6

[77] Ferrero S , Wittoek R, Allado E et al Methotrexate treatment in hand osteoarthritis refractory to usual treatments: a randomised, double-blind, placebo-controlled trial. Semin Arthritis Rheum 2021;51:831–8. 34157578 10.1016/j.semarthrit.2021.04.016

[78] Wittoek R , Verbruggen G, Vanhaverbeke T, Colman R, Elewaut D. RANKL blockade for erosive hand osteoarthritis: a randomized placebo-controlled phase 2a trial. Nat Med 2024;30:829–36. 38361122 10.1038/s41591-024-02822-0 PMC10957468

[79] Cai G , Laslett LL, Thompson M et al Effect of intravenous zoledronic acid on total knee replacement in patients with symptomatic knee osteoarthritis and without severe joint space narrowing: a prespecified secondary analysis of a two-year, multicenter, double-blind, placebo-controlled clinical trial. Arthritis Rheumatol 2024;76:1047–53. 38369770 10.1002/art.42831

[80] Watt FE. Hand osteoarthritis, menopause and menopausal hormone therapy. Maturitas 2016;83:13–8. 26471929 10.1016/j.maturitas.2015.09.007

[81] Richette P , Corvol M, Bardin T. Estrogens, cartilage, and osteoarthritis. Joint Bone Spine 2003;70:257–62. 12951307 10.1016/s1297-319x(03)00067-8

[82] Dennison EM. Osteoarthritis: the importance of hormonal status in midlife women. Maturitas 2022;165:8–11. 35841775 10.1016/j.maturitas.2022.07.002

[83] National Institute for Health and Care Excellence. Menopause: diagnosis and management. NG23. London: National Institute for Health and Care Excellence, 2015.

[84] Williams JAE , Chester-Jones M, Minns Lowe C et al Hormone replacement therapy (conjugated oestrogens plus bazedoxifene) for post-menopausal women with symptomatic hand osteoarthritis: primary report from the HOPE-e randomised, placebo-controlled, feasibility study. Lancet Rheumatol 2022;4:e725–37. 36341025 10.1016/S2665-9913(22)00218-1 PMC9620575

[85] Gulati M , Dursun E, Vincent V, Watt FE. The influence of sex hormones on musculoskeletal pain and osteoarthritis. Lancet Rheumatol 2023;5:e225–38. 10.1016/S2665-9913(23)00060-7 38251525

[86] Bliddal H , Bays H, Czernichow S et al Once-weekly semaglutide in persons with obesity and knee osteoarthritis. N Engl J Med 2024;391:1573–83. 10.1056/NEJMoa2403664 39476339

[87] Meurot C , Jacques C, Martin C et al Targeting the GLP-1/GLP-1R axis to treat osteoarthritis: a new opportunity? J Orthop Translat 2022;32:121–9. 35280931 10.1016/j.jot.2022.02.001 PMC8888891

[88] Hatzikotoulas K , Southam L, Stefansdottir L et al Translational genomics of osteoarthritis in 1,962,069 individuals. Nature 2025;641:1217–24. 40205036 10.1038/s41586-025-08771-z PMC12119359

[89] Minikel EV , Painter JL, Dong CC, Nelson MR. Refining the impact of genetic evidence on clinical success. Nature 2024;629:624–9. 10.1038/s41586-024-07316-0 PMC11096124 38632401

[90] Ottenhoff JSE , Spaans AJ, Braakenburg A et al Joint distraction for thumb carpometacarpal osteoarthritis: 2-year follow-up results of 20 patients. J Wrist Surg 2021;10:502–10. 34881105 10.1055/s-0041-1728806 PMC8635830

[91] Paskins Z , Farmer CE, Manning F et al Research priorities to reduce the impact of musculoskeletal disorders: a priority setting exercise with the child health and nutrition research initiative method. Lancet Rheumatol 2022;4:e635–45. 36275038 10.1016/S2665-9913(22)00136-9 PMC9584828

[92] Karantana A , Davis T, Kennedy D et al Common hand and wrist conditions: creation of UK research priorities defined by a James Lind Alliance Priority Setting Partnership. BMJ Open 2021;11:e044207. 10.1136/bmjopen-2020-044207 PMC8006829 33771825

[93] Kloppenburg M , Boyesen P, Visser AW et al Report from the OMERACT Hand Osteoarthritis Working Group: set of core domains and preliminary set of instruments for use in clinical trials and observational studies. J Rheumatol 2015;42:2190–7. 26136489 10.3899/jrheum.141017

